# Interaction of haemin with albumin-based macroporous cryogel: Adsorption isotherm and fluorescence quenching studies

**DOI:** 10.3389/fbioe.2022.1072153

**Published:** 2022-11-28

**Authors:** Solmaz Hajizadeh, Cedric Dicko, Leif Bülow

**Affiliations:** Division of Pure and Applied Biochemistry, Department of Chemistry, Lund University, Lund, Sweden

**Keywords:** haemin, albumin, macroporous network, double-layer cryogel, adsorption isotherm, binding sites

## Abstract

Albumin-based cryogels for capturing haemin were synthesised by crosslinking different biomolecules, bovine serum albumin (BSA) and ovalbumin (OVA). The impact of the protein and coupling agent concentrations on cryogel’s mechanical properties, swelling ratios and polymerisation yields, as well as autoclaving as a post-treatment on the cryogel, were studied. We found that BSA (50 mg/ml) and the crosslinker (*N*-(3-dimethylaminopropyl)-*N′*-ethylcarbodiimide hydrochloride, 46 mg/ml) formed a cryogel with optimum physical characteristics at a comparatively low protein concentration. The cryogel’s mechanical stability was increased using a double-layer cryogel approach by crosslinking the BSA proteins at subzero temperature inside an acrylamide and hydroxyethyl methacrylate premade cryogels. Batch binding and kinetic adsorption isotherms of haemin on the cryogels were assessed to evaluate their binding capacity toward the porphyrin molecule. The results showed that single-layer cryogels (BSA and OVA) had a higher capacity (∼0.68 mg/ml gel) and higher reaction rate constant towards haemin adsorption than double-layer gels. In contrast, the double-layer cryogels had higher mechanical strength than single-layer gels. The experimental results suggested that the cryogels followed the Freundlich model and the pseudo-second-order isotherm for batch adsorption and kinetics, respectively. The interaction between haemin and the gels was studied by fluorescence quenching. We found between 1.1 and 1.6 binding sites for different cryogels.

## Introduction

Human haemoglobin (Hb) comprises four subunits, two alpha and two beta units, each carrying one haem molecule (Ahmed et al., 2020). Haem, Fe^2+^ protoporphyrin IX is crucial in transporting oxygen in the bloodstream. The interaction between haem and globin is stable under physiological conditions, and the iron atoms are kept in the ferrous state by intracellular methemoglobin reductases and $O_{2}^{{}^{\circ}-}$ (Hargrove et al., 1997). However, under the autooxidation effect, Fe^2+^ transforms to Fe^3+^ and changes to methemoglobin (Hebbel et al., 1988). Under such conditions, haem can escape from its protein cavity. Released haem (haematin) is an inflammatory-inducing molecule due to its ability to generate reactive oxygen species (ROS). ROS can alter membrane permeability and human erythrocytes (Graça-Souza et al., 2006) and is recognised as a damage-associated molecular pattern, DAMP. References to haem cytotoxicity can be found elsewhere (Wagener et al., 2001; Aich et al., 2015). In a functioning body, free haem uptake is facilitated by specific serum transport proteins (globin, albumin and haemopexin) and distributed according to their concentrations, affinities for haem, and rates of clearance from the circulation (Morgan et al., 1976). However, in patients (Fontanellas et al., 1994), blood transfusion bags (Vijayan et al., 2022) or when designing haemoglobin-based oxygen carriers (HBOCs) (Bialas et al., 2019), the free haem ions should be removed by other means and methods, such as filtration or dialysis (Fontanellas et al., 1994; Coronel et al., 2003), to minimise their side effects. However, these methods have a few limitations, such as fouling and low efficiency when dealing with suspensions and complex mixtures. Thus, an alternative solution is needed.

In this study, a new class of materials, known as cryogels, was introduced to deplete haem from a solution or suspension. Cryogel (from the Greek word kryos, meaning ice) is a subcategory of hydrogels. The gel material results from the cryotropic gelation (cryogelation) technique that allows forming macroporous polymeric networks with interconnected channels and controlled porosities (Plieva et al., 2004). During cryogelation, the polymer/monomer solution is frozen at a low temperature to create unfrozen and frozen phases. The polymerisation occurs in the unfrozen domains where the concentrations of the chemicals are remarkably high, and the ice crystals (frozen phase) act as porogens. The final product is a permanent macroporous structure where the geometry of the ice crystals determines its final pore size (Hajizadeh et al., 2012). The advantage of cryogels over hydrogels or other polymeric membranes is their large interconnected pores, which allow efficient mass transfer and good flow properties. Since the introduction of cryogels in the 1970s, many different synthetic monomers/polymers, biological molecules and particles have been used to form cryogels (Aragão Börner et al., 2014; Agarwal et al., 2021; Jones et al., 2021). These materials are found in many different applications ranging from separation and purification (Hajizadeh et al., 2021) to biomedical applications (Henderson et al., 2013) and environmental applications (Jain and Kumar, 2013; Berillo et al., 2021).

Porous albumin-based cryogels are popular in various biomedical applications due to their biocompatibility and biodegradability (Kirsebom et al., 2013). In this study, serum albumin protein from bovine and ovalbumin were crosslinked to form a macroporous cryogel for capturing free haemin molecules from an aqueous solution. The suggested material is a potentially good candidate in biomedical and biotechnology applications for removing haem frommixtures. However, the drawback of these materials is their low mechanical stability, making them soft, unsteady and challenging to handle. This issue has been addressed in this study by forming a double-layer cryogel. Double-layer cryogel refers to forming one type of cryogel inside another premade macroporous gel. The binding capacity and kinetics of the different gels were evaluated and discussed, as well as the number of their binding sites.

## Materials

Bovine serum albumin (BSA ≥98%, heat shock fraction), haemin, ovalbumin (OVA ≥98%), acrylamide (Am), *N*,*N*′-methylenebis (acrylamide) (MBAm, 99%), *N,N,N′,N′*-tetramethylethylenediamine (TEMED ≥99%), ammonium persulfate (APS ≥98.0%), 2-hydroxyethyl methacrylate (HEMA ≥99%), sodium hydrogen phosphate, sodium dihydrogen phosphate, sodium hydroxide (NaOH), and *N*-(3-dimethylaminopropyl)-*N′*-ethylcarbodiimide hydrochloride (EDC) were all purchased from Merck (Sigma-Aldrich, Burlington, MA, United States) and applied as received.

## Methods

### Single-layer albumin-based cryogel synthesis

BSA cryogels were synthesised by crosslinking the protein using EDC, as reported elsewhere (Rodionov et al., 2016). Briefly, different masses of BSA powder were dissolved in water to prepare some solution at various concentrations (Table 1). The solutions were cooled in an ice bath. The appropriate amount of crosslinker (EDC) was then added to the BSA solutions and stirred using a magnet while standing in an ice bath. Aliquots (0.25 ml) of the solutions were transferred into precooled glass tubes (i.d. 7 mm) and frozen at −16°C in a cryostat for 2 days. Once the reaction was completed, the gels were thawed at room temperature and washed with water to remove unreacted chemicals.

**TABLE 1:** Formation conditions, polymerisation yield, swelling rate and mechanical properties of the BSA cryogel.

BSA (mg/ml)	EDC (mg/ml)	Molar ratio COOH/ECD/NH_2_	Yield (%)	Swelling rate (%)	Hardness (N)	Compressibility (N mm)
30	37	1: 5.5: 0.6	51.50 ± 1.4	15.78 ± 1.0	—	—
40	37	1: 4: 0.6	46.80 ± 1.7	20.85 ± 3.2	0.94 ± 0.16	0.51 ± 0.13
50	31	1: 2.4: 0.6	40.93 ± 2.5	20.52 ± 1.6	3.55 ± 0.89	1.94 ± 0.47
50	37	1: 3.2: 0.6	52.34 ± 0.9	14.35 ± 2.6	1.1 ± 0.02	0.61 ± 0.02
50	46	1: 3.9: 0.6	53.73 ± 1.3	15.43 ± 1.9	1.49 ± 0.06	0.27 ± 0.04
50	56	1: 4.6: 0.6	45.37 ± 6.8	16.84 ± 2.8	5.25 ± 4.38	2.9 ± 2.4
50	62	1: 5.3: 0.6	44.04 ± 0.4	12.18 ± 1.5	1.41 ± 0.83	0.76 ± 0.45

OVA cryogels were prepared with the same procedure mentioned above, except that the OVA molecule was used instead of BSA proteins. The BSA and OVA cryogels were kept in water at 4°C and henceforth referred to as BSA-CG and OVA-CG, respectively.

### Double-layer cryogel synthesis

To synthesise the double-layer cryogels, Am or HEMA monomers were used as the main building block for the primary cryogel. The preparation of cryogel using radical polymerisation has been reported elsewhere (Hajizadeh, 2012). The monomer concentration of monomers was kept at 6%, and the molar ratio between Am (or HEMA) and MBAm was 6: 1. An activator, TEMED (1% of total monomer volume), was added to the mixture. Then, the solution was degassed under negative pressure to remove any soluble oxygen while the solution was cooled in an ice bath for a minimum of 5 min. Ammonium persulfate (APS) solution (500 μL, 1% of total monomer volume) as an initiator was added to the monomer mixture in the final step. Aliquots (0.25 ml) of the solution were transferred into glass tubes (i.d. 7 mm) and frozen at −12°C in a liquid cryostat overnight. The gels were thawed at room temperature, washed with water to remove all the unreacted monomers, and dried at 60°C. The gels are henceforth referred to as Am-CG and HEMA-CG for the Am and HEMA cryogels, respectively.

The procedure to prepare the second layer inside the primary cryogel is similar to the formation of the single-layer cryogel, as mentioned earlier, with a few minor changes. A dried Am-CG (HEMA-CG) was placed inside glass tubes (i.d. 7 mm). BSA and EDC concentrations were kept at 50 and 46 mg/ml, respectively. The cooled mixture of protein and crosslinker (0.25 ml) was added to the glass tube over the dried Am-CG (or HEMA-CG). After the gel absorbed the solution, the glass tube was sealed and placed inside the cryostat for 2 days at −16°C. The new cryogels are referred to as Am-BSA-CG (HEMA-BSA-CG) based on the type of the first layer of cryogel. The gels were kept in water at 4°C.

### Characterisation of cryogels

The polymerisation yield for each cryogel was calculated based on the equation below:

$$Y\left(\%\right)=\frac{W_{1}}{W_{0}}\times 100$$ (1)

where *W*_*0*_ and *W*_*1*_ are the theoretical weight and the measured dry weight of the cryogels, respectively. All gels were dried in an oven at 60°C overnight.

The swelling degree was calculated using Eq. 2:

$$S=\frac{W_{2}-W_{1}}{W_{1}}$$ (2)

where *W*_*1*_ and *W*_*2*_ are the weights of the dried and swollen cryogels, respectively. The gels were swollen in a water solution, and the excess water was blotted away using filter papers.

Scanning electron microscopy (SEM) was used to study the morphology of the macroporous gels. The middle section of the cryogel was cut transversally into thin discs (1–2 mm thick) and freeze-dried. The discs were sputtered with gold: palladium (40:60, to a thickness of 15 nm) and imaged using a JEOL JSM-80 5600 LV microscope (Tokyo, Japan).

Thermo Nicolet 6700 FTIR spectrometer equipped with a single bounce diamond ATR and a DTGS detector (Thermo Fisher-Scientific Inc. United States) was used to study Fourier-transform infrared spectroscopy (FTIR) spectra of the different cryogels. Different cuts were made from each gel’s top, middle and bottom sections for FTIR analysis.

A texture analyser (XT2i, Stable Micro Systems, Godalming, England) and Exponent v.5.0.9.0 software (Godalming, England) were used to assess the mechanical stability and the data analysis, respectively. The texture analyser had a 2 kg load cell, and the compressive force was applied at a constant rate of 0.5 mm/s. The wet cryogels (stored in a water solution overnight) were placed on a metal plate and compressed up to 50% of their original height. The compression test was performed in triplicate. Young’s modulus was extracted as follows (Eq. 3):

E=σε=FA0∆ll0 (3)

where *E* is Young’s modulus (Pa), *F* is the force applied to the object (N), *A*_*0*_ is the cross-sectional area of the cryogel on which the force is applied (m^2^), *Δl* is the change in length under compression, and *l*_*0*_ is the original height of the object. The hardness and compressibility of the material can also be extracted from force-time graph data. Hardness is a force required to attain a given deformation, and compressibility explains the work required to deform the product under the first compression cycle (Jones et al., 1997).

To study the effect of sterilisation on the protein cryogel characterisation, wet BSA-CG was autoclaved for 20 min at 121°C and then refrigerated.

### Batch binding adsorption

For the batch adsorption experiment, a stock solution of haemin (6 mg/ml) was prepared in phosphate buffer (0.1 M, pH 7.4). Haemin is soluble in alkaline solutions, e.g., NaOH and organic solvents such as dimethyl sulfoxide (DMSO). The powder was first dissolved in a low volume (∼1 ml) of NaOH solution (0.01 M) to make the stock solution and then diluted with phosphate buffer (0.1 M, pH 6). If needed, the final pH was adjusted to 7.4 by adding NaOH droplets (6 M).

Two millilitres of different haemin concentration solutions (0.1, 0.2, 0.4, 0.8, 0.16, 0.32, 0.65, 1.3 mg/ml) were prepared from the stock solution using phosphate buffer (0.1 M, pH 7.4) in Eppendorf tubes. In each tube, a wet cryogel was added and gently shaken on a rocking table at room temperature for 24 h. After binding, the supernatants were measured by a NanoPhotometer Pearl ultraviolet-visible (UV-Vis) spectrophotometer (Munich, Germany) at 390 nm wavelength to determine the remaining haemin concentration in the solution (Ɛ_390_ = 34.4×10^4^ M^−1^ cm^−1^). To calculate the nonspecific binding, each gel was washed with 2 ml carbonate buffer (0.1 M, pH 9) overnight on a rocking table at room temperature. The amount of the adsorbed haemin on the gel was calculated by the depletion method using Eq. 4:

$$Q=\frac{\left(C_{i}-C_{eq}-C_{w}\right)V}{v}$$ (4)

where Q is the capacity of the cryogel (mg of haemin/mL of gel), *C*_*i*_, *C*_*eq,*_ and C_w_ are the haemin initial, equilibrium concentrations in solution and concentration in wash solution (mg/ml), respectively. *V* is the volume of the haemin solution (ml) in contact with the cryogel, and *v* is the volume of the cryogel (ml).

Time-resolved adsorption was performed on different cryogels with a haemin solution (0.2 mg/ml). The cryogel was left in contact with the solution for 1, 2, 5, 10, 20, 30, 40, 50, 60, 120, 180, 240, 300, 360, 960, 1,020, 1,080, 1,140, 1,200, 1,260, 1,320, 1,380 and 1,440 min (24 h) before the haemin concentration in solution was measured using the above molar adsorption coefficient. One cryogel (0.25 ml) was added to the haemin solution in a separate tube for each data point.

### Binding site

**FIGURE 1:**
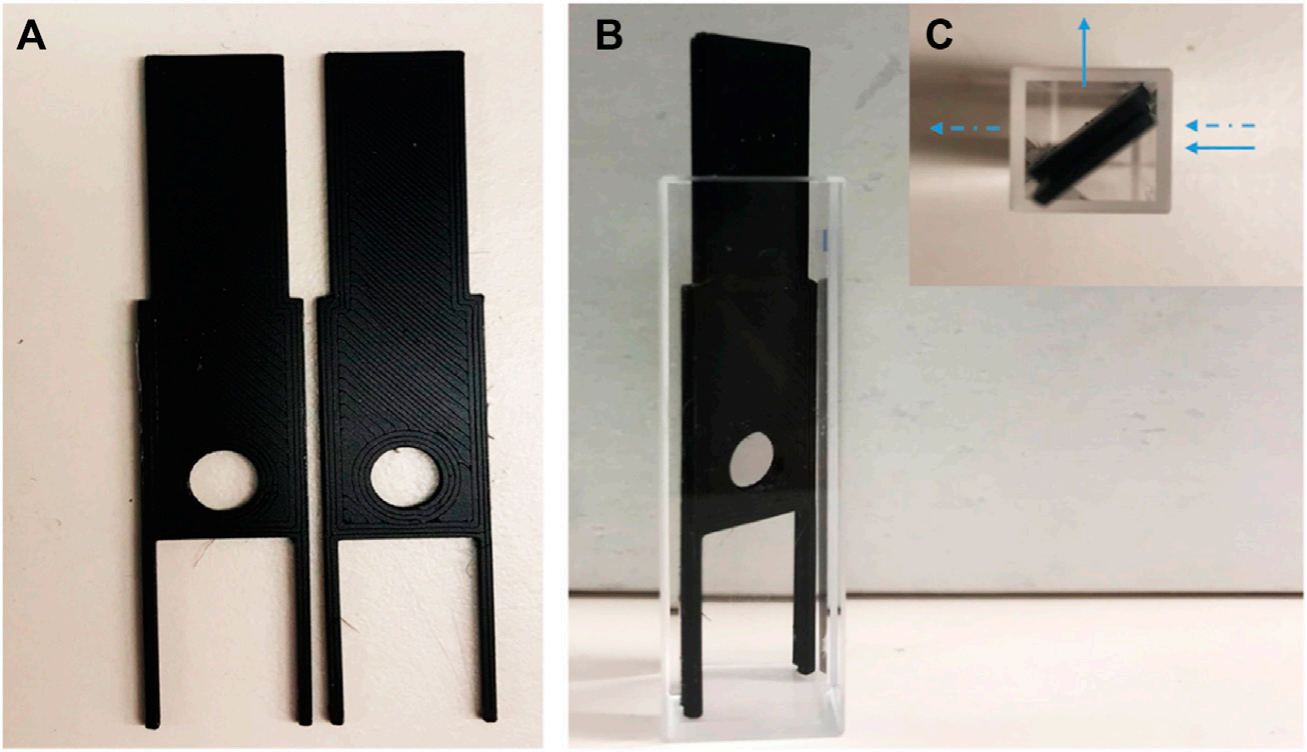
Digital image of **(A)** plastic holders; **(B)** plastic holder inside cuvette with 45° (side view); **(C)** the holders in the cuvette (top view). The arrows show the direction of fluorescent light (←) and UV-Visible light (⇠).

The following procedure was used to determine the number of binding sites of the crosslinked proteins in single- and double-layer cryogels. The gels were frozen at −20°C overnight, and then thin film layers (<< 1 mm thickness, approximately 5–9 mg dried weight) were manually cut with a sharp surgical blade. The film was held between two plastic parts with a hole (Figure 1) and inserted diagonally (45°) into a 10-mm quartz cuvette. The plastic holder was custom-designed, and 3D-printed in-house on a Prusa MK3 fused filament printer (Prague, Czech Republic) with polylactic acid. The fluorescence was excited at 295 nm, and the emission spectrum was recorded between 300 and 500 nm. Cryogel fluorescence emissions with and without haemin were measured using Cary Eclipse Spectrofluorometer, Agilent Technologies, Frankfurt, Germany. For the inner filter effect corrections, the cryogel films were placed inside the same holders and read by a UV-Vis spectrophotometer. However, due to the thickness of the film, no data could be extracted.

Each thin film of cryogels was placed in separate haemin solutions (2 ml) with different concentrations (1.2, 2.4, 3.5, 4.7, 5.8, 6.9, 8.02 and 9.08 μM) and gently shaken on a rocking table overnight at room temperature. Then, the films were mounted in the plastic holder to measure their fluorescence emission spectrum. The estimation of the number of binding sites was done by fitting the following equation:

$$\log\frac{F_{0}-F}{F}=\log K_{a}+n\;\log\left[Q\right]$$ (5)

where K_a_ is the binding constant, n denotes the number of binding sites and [Q] is the final concentration of the quencher (haemin).

## Results and discussion

### Albumin-based cryogel’s characterisation

BSA (molecular mass of approximately 66.4 kDa) carries 583 amino acid residues, including 35 cysteines, 99 residues of aspartic and glutamic acids (in total), and 60 lysine units (Peters, 1995). The presence of β- and γ-carboxyl groups and ε-amino groups in the protein makes it possible to crosslink them in solution by applying coupling agents, such as carbodiimide, where inter and intrachain peptide bond formation is responsible for the gel matrices (Topală et al., 2014).

Different carboxyl and amino groups in BSA have different steric accessibilities for inter (intra) molecular crosslinking. Thus, to improve cryogel mechanical properties, various concentrations of BSA and the coupling agent were selected to conclude which concentration would give the most suitable gel network with the right physical properties for this work (Table 1). Lozinsky and his team explained the mechanism of the crosslinking agent and the BSA elsewhere. They estimated the molar ratio between the carboxyl group, coupling agent and the amine group (COOH: EDC: NH_4_) to be from 1:0.8:0.6 to 1:5.5:0.6 range (Rodionov et al., 2016).

**FIGURE 2:**
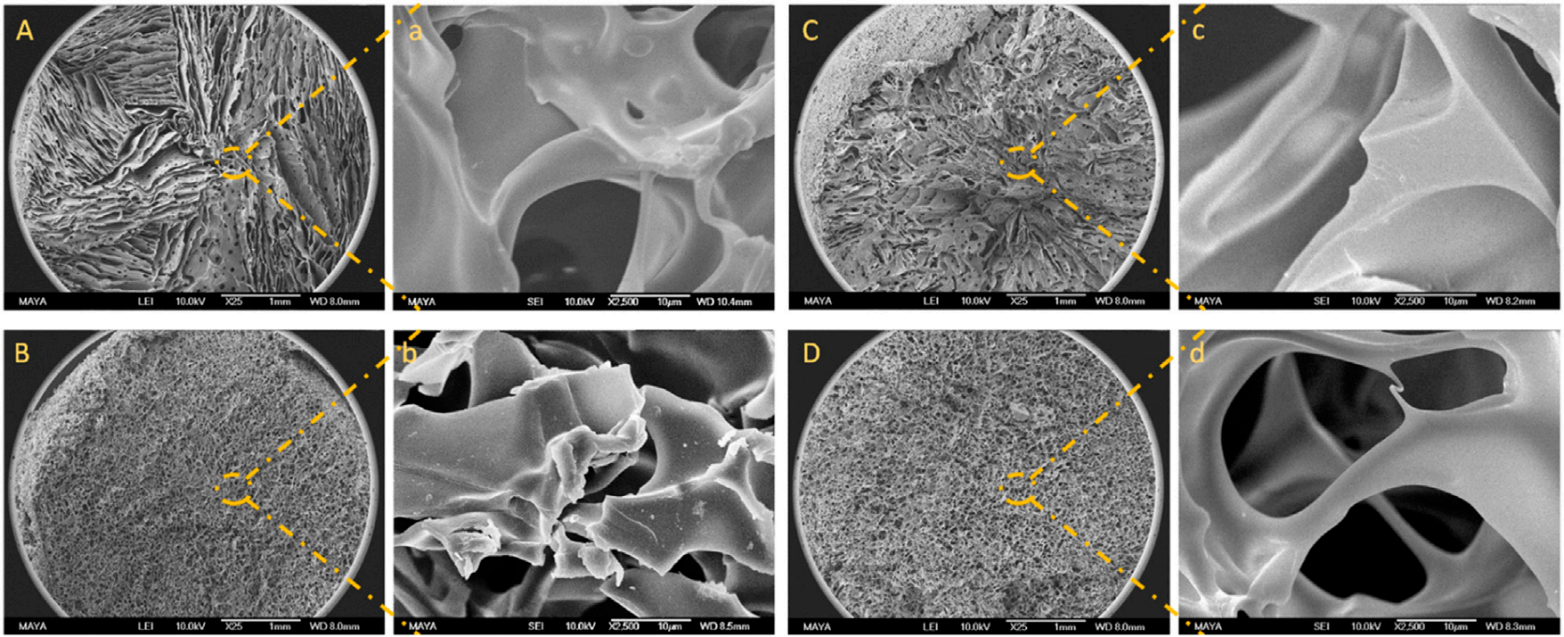
SEM images of BSA-CG with BSA (50 mg/ml) and EDC: **(A)** 37 mg/ml; **(B)** 46 mg/ml; **(C)** 56 mg/ml, and **(D)** 62 mg/ml. Images a, b, c and d are the high magnification of images A, B, C and D, respectively. The scale bars are 1 mm and 10 μm for low- and high-magnification images, respectively.

The macroporous structure of BSA-CG was assessed by scanning electron microscopy (Figure 2; Supplementary Figure S1). From Figure 2A, the gel cross-section seems to have divided sections, and in each section, the direction of the wall (pores) was more aligned, while in Figures 2B,D, the formation of the pores seemed to be more random. The differences in alignment and configuration of the cryogel’s structure depend on the freezing conditions, such as the rate of freezing, presence of impurity (in this case, protein and the coupling agent concentrations), temperature, and formation of ice crystals’ nuclei (Kirsebom et al., 2010). Based on the SEM images (Supplementary Figure S1) and the ImageJ (1.53 C version), the average pore size for the three cryogels (A, B and C) with different protein concentrations is 30, 44 and 37 μm, respectively. The difference originated from the freezing procedure and the formation of ice crystals in the material (Plieva et al., 2004). The pore size distribution inside a dry cryogel was studied and reported using mercury porosimetry analysis (Hajizadeh et al., 2010).

Based on the polymerisation yield, swelling ratio and mechanical strength of the BSA-CGs, the most suitable gel formation condition was selected for further study. BSA (30 mg/ml) stands in a group with a high polymerisation yield and average swelling ratio. However, the gel was very soft and fragile as it collapsed under compression. Compared to the other cryogels, 40 mg/ml BSA had the highest swelling ratio but the lowest mechanical strength. The BSA-CG formed with 50 mg/ml BSA and 56 mg/ml EDC displayed the highest mechanical strength among the other cryogels. The result of the higher mechanical strength was explained by increasing the number of covalent bonds between the crosslinker and the protein during polymerisation. The polymerisation yield and the swelling ratio were in the average range compared to the other cryogels in Table 1. However, the repeatability of the experiment was low and resulted in a significant difference between the mechanical strength of the cryogels (Table 1; Supplementary Figure S2). Thus, 50 mg/ml and 46 mg/ml were selected as reference concentrations for BSA and EDC, respectively, for the rest of this work. The same biomolecule concentrations and the coupling agent was used for OVA-CG, Am-BSA-CG and HEMA-BSA-CG.

One of the disadvantages of biomacromolecule-based cryogels is that the resulting gels are often soft and require less energy to break than synthetic equivalents (Panahi and Baghban-Salehi, 2019). Different strategies may be applied to improve this physical shortcoming, such as forming composite cryogels by adding additional monomers during polymerisation to strengthen the final material (Gonzalez and Alvarez, 2014; de Lima et al., 2018) or cryogelation inside an external carrier. For the latter, plastic holders (Le Noir et al., 2009; Önnby et al., 2010) or a premade cryogel (Sedlačík et al., 2020). In this study, the robustness of the albumin-based cryogel was improved by forming a cryogel inside a primary cryogel. Acrylamide and HEMA monomers were selected as the building blocks for the first cryogel layer. Polyacrylamide and pHEMA cryogels are well studied and have shown great potential in different applications (Carvalho et al., 2014; Ali et al., 2018). However, their application in the biomedical field is limited due to the risk of toxicity of free Am monomers (Zamani et al., 2017; Cantrell and McDougal, 2021). Thus, a poly (HEMA) cryogel was designed as an alternative. HEMA is compliant with medical applications (Zare et al., 2021).

**FIGURE 3:**
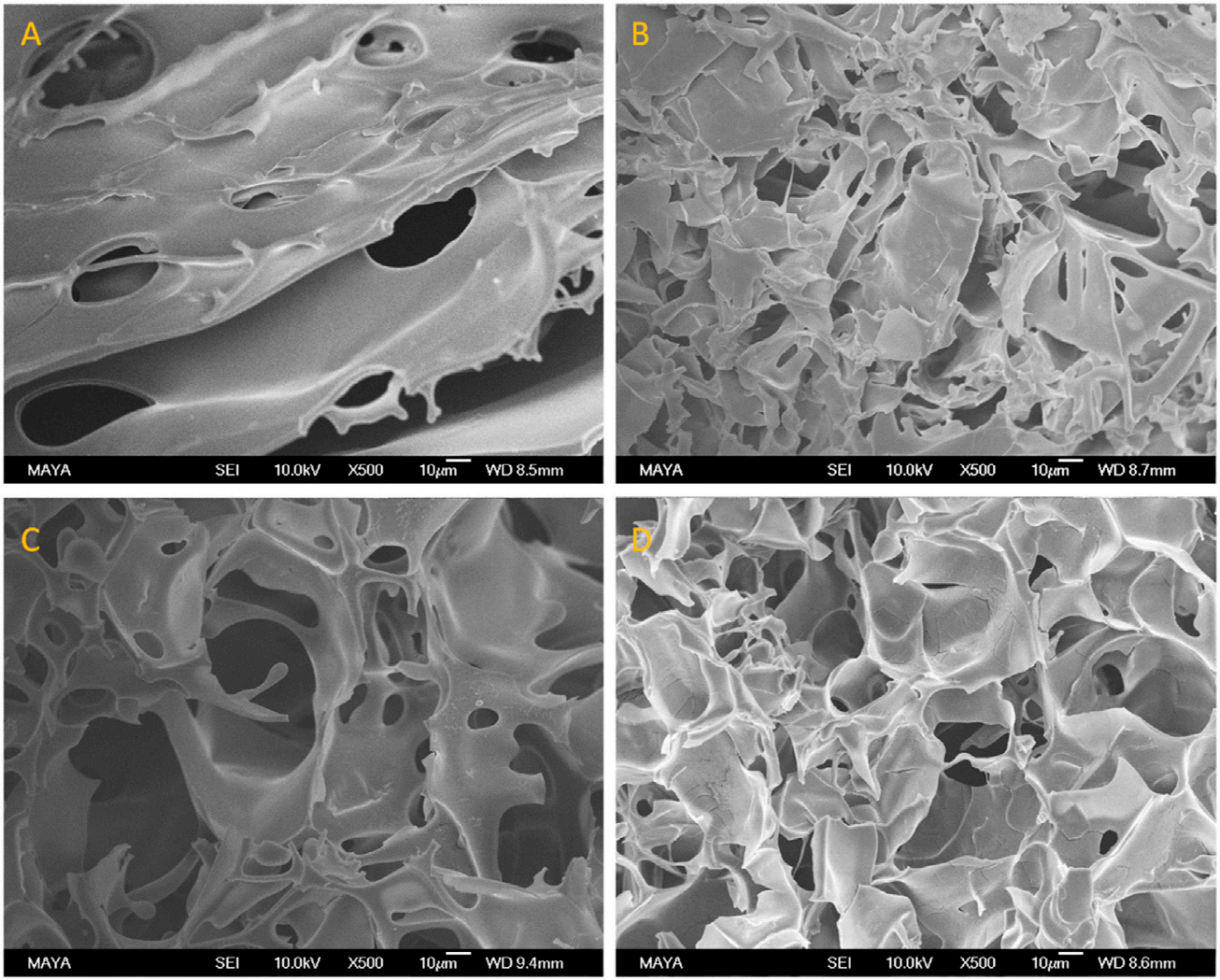
SEM images of **(A)** HEMA-CG, **(B)** HEMA-BSA-CG, **(C)** Am-CG, and **(D)** Am-BSA-CG. The scale bar is 10 μm.

Figure 3 shows the Am-CG and HEMA-CG structures before and after introducing the albumin-based second crosslinked network. The images display the macroporous structure of the cryogel after the second cryogelation process. The average pore size measured by ImageJ based on the SEM image (Figures 3B,D) was 42 and 37 μm for Am-BSA-CG and HEMA-BSA-CG, respectively.

The ultimate goal of this study is to design materials for biomedical applications, so being able to sterilise them and retain their initial properties is crucial. BSA-CG was autoclaved, and its physical and chemical properties were compared with those before autoclaving. Other methods for sterilisation, e.g., ionising radiation and liquid chemical sterilisation (e.g. ethylene oxide), can be applied and may not dramatically affect soft materials (Berovic, 2005). However, this work did not prioritise evaluating different sterilisation methods on the designed materials. After autoclaving, the size of the cryogel was slightly shrunk (∼1 mm in diameter), and its colour changed to beige-yellow due to protein oxidation (Supplementary Figure S3). The SEM images of the albumin-based cryogel before and after autoclaving illustrate that the macroporous network was undamaged during the post-treatment. The results indicated that the covalent bonds between the biomolecules and the crosslinker are stable at the macro scale (Supplementary Figure S4). However, at higher magnification, the initially smooth surface of the albumin-based cryogels showed wrinkles after autoclaving compared to the control cryogel, Am-CG (Supplementary Figure S4B,D). Under such extreme operating conditions, dehydration was expected for the two types of cryogel (protein and synthetic based). However, denaturation of the biomolecules under high temperature and pressure seemed to have more impact on the surface morphology.

The mechanical properties of the cryogels are summarised in Table 2; Supplementary Figure S5. The linear range between 5 and 30% of strain was considered the elastic region for calculating the elasticity by using Young’s modulus (E) and comparing the data for all the different cryogels. The double-layer cryogels had a higher modulus than the single albumin-based cryogels, explained by the presence of the second polymeric layer, providing a denser gel with more crosslinks. The gain in mechanical strength allowed better handling of the cryogel.

**TABLE 2:** The mechanical properties of the cryogels.

	BSA-CG	OVA-CG	Am-BSA-CG	HEMA-BSA-CG	Autoclaved BSA-CG
E (kPa)	1.93 ± 0.08	1.02 ± 0.17	5.01 ± 0.82	6.82 ± 3.03	11.51 ± 2.64
Hardness (N)	0.47 ± 0.15	0.27 ± 0.19	0.42 ± 0.14	0.45 ± 0.38	0.12 ± 0.02
Compressibility (N.mm)	0.54 ± 0.13	0.41 ± 0.12	1.23 ± 0.45	1.27 ± 0.76	0.35 ± 0.08

### Batch binding adsorption

**FIGURE 4:**
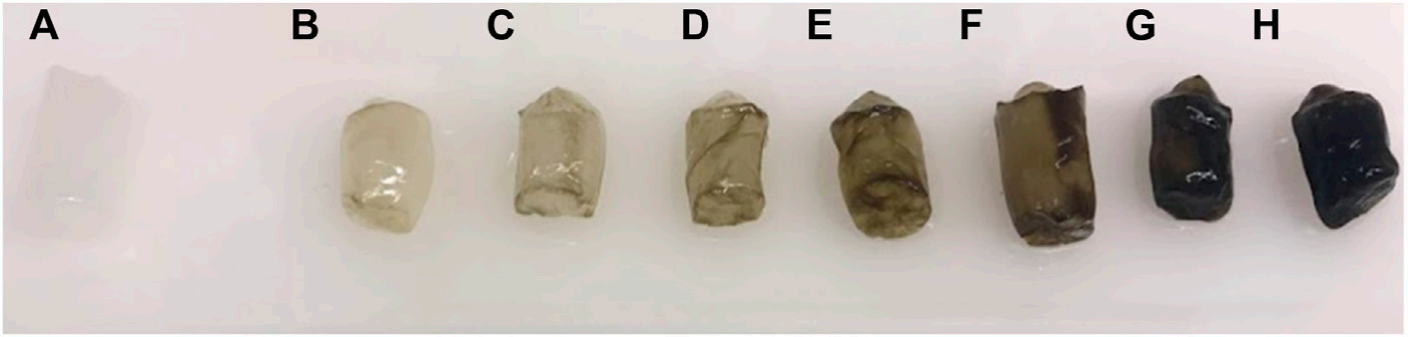
Digital image of BSA-CG before and after interaction with different concentrations of haemin solutions: **(A)** 0; **(B)** 0.004; **(C)** 0.008; **(D)** 0.016; **(E)** 0.032; **(F)** 0.065; **(G)** 0.13 and **(H)** 0.26 mg/ml haemin in 0.1 M phosphate buffer pH 7.4.

Figure 4 displays a digital image of BSA-CG before and after contact with different concentrations of haemin solution. The dark green colour of the cryogel in the image indicates a high accumulation of the porphyrin molecule as a result of both specific and nonspecific interactions (Makarska-Bialokoz, 2018). The green colour stemmed from the oxe-bridge Fe-O-Fe dimer of haemin in solution (Brown et al., 1969).

**FIGURE 5:**
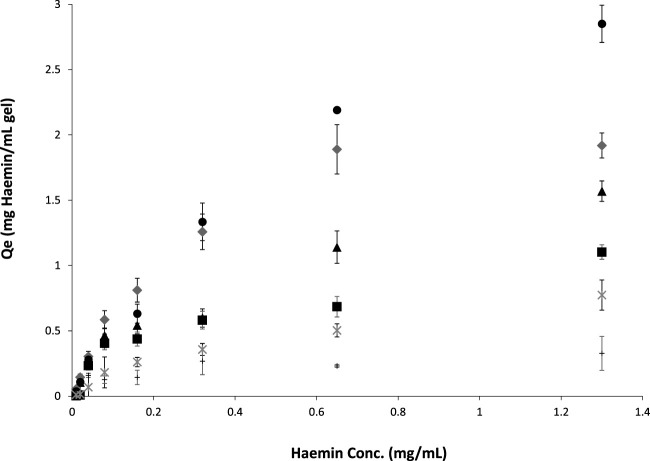
Haemin adsorption isotherms on different cryogels at room temperature for 24 h. ●: OVA-CG; ♦: BSA-CG; ▲: Am-BSA-CG; ■: HEMA-BSA-CG; *: autoclaved BSA-CG and +: Am-CG.

Figure 5 shows the binding adsorption of haemin on different types of cryogels. The number of tryptophan residues plays an essential role in the interaction and adsorption of the porphyrin molecule (Yadav et al., 2012; Makarska-Bialokoz, 2018). OVA and BSA have 3 and 2 tryptophan residues, respectively (Ishimaru et al., 2010; Raut et al., 2014). However, only one residue is available on the surface of these proteins, which can interact with the target (Makarska-Bialokoz, 2018) for the free proteins in the solution. Crosslinked proteins in a network may have different conformations (secondary and tertiary structures) and surface accessibility. For example, the crosslinking-induced protein opening could expose the inner tryptophan residues. In addition, the nonspecific binding of the porphyrin molecule with the polymeric network is another factor to consider in batch adsorption. For the single-layer cryogel, the nonspecific adsorption can be related to the accumulation of the target molecules inside the micro and macro pores or their physical interaction with each other, which can be calculated from the washing step using carbonate buffer. However, in the double-layer cryogel, the premade cryogel can interact physically with the porphyrin molecules in addition to the other nonspecific interactions. Therefore, a control experiment was conducted to study the batch-binding adsorption of haemin on Am and HEMA-CGs. The data revealed that both synthetic cryogels have low-affinity interaction with the target molecules (Figure 5, data related to HEMA-CG is not shown). The results showed that OVA-CG has a slightly higher binding capacity towards haemin than the other cryogels (Figure 5).

We fitted and compared the adsorption isotherm using Langmuir, Freundlich and Temkin models. A F-test (*p* < 0.05) comparison of the models suggested that the Freundlich model was best suited for all cryogels except for BSA-CG autoclaved (Supplementary Figure S6; Table 3).

**TABLE 3:** The Freundlich adsorption isotherm for haemin adsorption on different types of protein cryogels.

	BSA-CG	OVA-CG	Am-BSA-CG	HEMA-BSA-CG	Am-CG	HEMA-CG
K_F_ (mg^1-1/*n*^mL^1/*n*^/mL)	2.27 ± 0.73	4.05 ± 0.51	1.39 ± 0.3	1.06 ± 0.43	4.85 ± 0.31	4.74 ± 0.11
1/n	0.24 ± 0.1	0.63 ± 0.05	0.31 ± 0.01	0.27 ± 0.07	0.29 ± 0.09	0.31 ± 0.07
R^2^	0.98	0.89	0.94	0.94	0.85	0.87

In the Freundlich model, surface adsorption is assumed heterogeneous. As the concentration of the adsorbate increases, its amount rises on the adsorbent surface. The Freundlich equation and its linearisation are as follows:

$$q_{e}=K_{F}{C_{e}}^{1/n}$$ (6)

$$In\;q_{e}=InK_{F}+1/nInC_{e}$$ (6a)

where KF is the Freundlich constant related to adsorption capacity, and n is the constant related to adsorption intensity. *K*_F_ is defined as the adsorption or distribution coefficient and represents the amount of adsorbate onto the adsorbent surface for a unit equilibrium concentration. The slope of 1/*n* ranging between 0 and 1 is a measure of adsorption intensity or surface heterogeneity. If the value approaches zero, the surface adsorption is more heterogeneous (Sathasivam and Mas Haris, 2010). The complex cryogelation process (e.g., dissolving the protein in water and not buffer, freezing at low temperature, crosslinking biomolecules) was expected to impact the protein structure and cause heterogeneous binding on the adsorption surface. Among the studied cryogels in this work, OVA-CG displays less heterogeneous surface adsorption when in contact with haemin based on the 1/n value. However, the goodness of fit was the lowest (Table 3). Autoclaved BSA-CG batch adsorption experimental data with haemin did not fit any of the well-known models, which may be explained by the oxidation of the protein molecules and densification of the cryogel during the treatment.

### Kinetics isotherm

**FIGURE 6:**
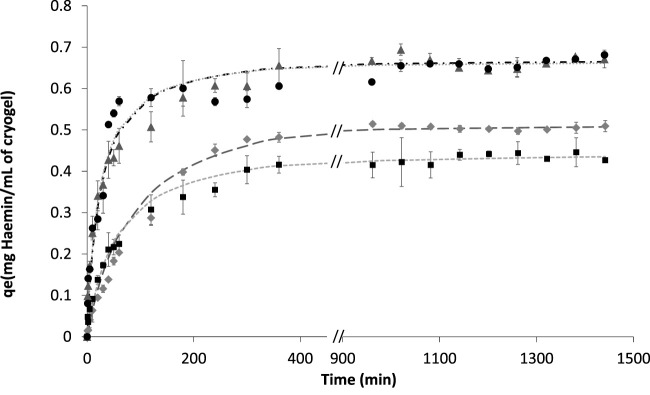
Fitting experimental data of haemin adsorption with initial concentrations of 0.2 mg/ml in 0.1 M phosphate buffer pH 7.4 for different cryogels. ▲ BSA-CG; ● OVA-CG; ◆ Am-BSA-CG and ■ HEMA-BSA-CG. Pseudo-second-order equation fitting with data: -- HEMA-BSA-CG; -.- BSA-CG; …. OVA-CG; _ _ _ Am-BSA-CG; No data available between 400 and 900 min.

The time-resolved binding studies were further performed to understand the interaction of haemin with the different cryogels. Different models were considered to fit the adsorption kinetic isotherm, e.g., the pseudo-first-order model, pseudo-second-order model, Elovich’s equations and intraparticle diffusion model (Weber and Morris, 1962) (Supplementary Figure S7). Based on a visual inspection of experimental data and confirmed by a model comparison using an F-test (*p* < 0.05), we concluded that the pseudo-second-order model is the better fit for different cryogels (Figure 6; Table 4).

**TABLE 4:** Pseudo-second-order parameters for the adsorption of haemin on albumin-based cryogels.

	BSA-CG	OVA-CG	Am-BSA-CG	HEMA-BSA-CG	Autoclaved BSA-CG
qe (mg/mL of gel)	0.69 ± 0.07	0.68 ± 0.03	0.59 ± 0.06	0.47 ± 0.03	0.43 ± 0.08
K2 (ml/mg h)	0.059 ± 0.003	0.064 ± 0.001	0.020 ± 0.007	0.039 ± 0.004	0.056 ± 0.01
R2	0.98	0.99	0.99	0.99	0.98

Figure 6; Supplementary Figure S8 show the kinetics studies for all the cryogels where the graphs showed two stages before reaching equilibrium; 1) a rapid increase based on the amount of haemin adsorbed as a function of time up to 60 min, and 2) the curve continues to increase with a much slower pace until reaching equilibrium for the initial haemin concentration (0.2 mg/ml in 0.1 M phosphate buffer pH 7.4). The two-step saturation curve may be explained by the free tryptophan residues available on the surface of the cryogel and those located inside the walls. In the latter case, diffusion of haemin through the walls is the limiting factor, while on the surface of the cryogel, the mass transfer is high between the available amino acid and free haemin (Supplementary Figure S8).

The time-resolved trace from the autoclaved BSA-CG (Supplementary Figure S9) was best fitted using the pseudo-second-order model. However, we observed a large discrepancy in the early stage of the binding. Visually, the non-autoclaved cryogel has a higher affinity (stronger colour) toward the porphyrin molecule (Supplementary Figure S10 digital image comparison of the autoclaved and non-autoclaved BSA-CGs in contact with haemin). The naked eye can detect the green colour changes in the non-autoclaved BSA-CG immediately after 1 min. In contrast, for the autoclaved cryogel, changes can be seen after a much longer time (Supplementary Figure S10).

As haemin can only be dissolved in a highly alkaline solution and some organic solvents, such as DMSO, removing the porphyrin molecule from cryogels is difficult. The strong binding of haemin to protein is used in affinity chromatography to separate and purify proteins. In those studies, to regenerate the column, one uses a high concentration of NaOH (1 M) and sodium dodecyl sulphate (SDS) solution (5%) (Tsutsui and Mueller, 1982; Jiang et al., 2017). This approach is incompatible with albumin-based cryogels and will destroy the structure of the gels, and the polymeric network will disintegrate during the process (Supplementary Figure S11). Additionally, in medical applications, one-time-use materials are preferred to minimise the risk of cross-contamination. Thus, regeneration and recycling of the designed material were not pursued further.

### Binding sites

There are reports in the literature investigating the mechanism of binding dye molecules to proteins using fluorescence quenching measurements (Barbero et al., 2009; Yadav et al., 2012). The interaction of haemin with BSA and the number of their binding sites was studied in detail elsewhere in an aqueous solution (Makarska-Bialokoz, 2018). However, none of these studies dealt with semi-solid materials. In this work, the protein is not a free molecule in a solution and, due to the crosslinking procedure, may even have a different structure inside the cryogel. FTIR spectra of the protein-based cryogels and pure BSA powder are plotted in Supplementary Figure S12. The results indicate that the secondary structure of the protein (amide I and II peaks at 1,650 and 1,540 cm^−1^, respectively) has changed after crosslinking compared to the free protein. The alteration was more noticeable in the double-layer cryostructured. Thus, different levels of accessibility of the haemin molecule to tryptophan can be expected. We studied the binding by fluorescence quenching of the tryptophan signal by haemin. First, the Stern–Volmer equation validity was confirmed for semi-solid gels. Before measuring the fluorescence emission, each film was incubated in its corresponding solution for 24 h on a rocking table to account for mass transfer limitation in the quenching process. As expected, the linearity was for F0/F vs*.* haemin concentration (Supplementary Figure S13). The linearity of the double-layer cryogels was lower than the single-protein cryogels, which was tentatively attributed to an inner filter effect. In addition, Am and HEMA polymers are hydrophilic (Domingues et al., 2013; Kordjazi et al., 2020). Thus, before cryogelation polymerisation, BSA can physically bind to the surface and within the premade cryogels, which could affect the protein positioning in the unfrozen region during cryogelation and, thus, accessibility of EDC to carboxylic and primary amine groups. In addition, even though Am and HEMA cryogels do not have any fluorescent properties (Supplementary Figure S14), their presence as an extra layer may not be neglected in collecting data from the fluorimeter.

The number of binding sites was determined by fitting the Stern–Volmer Eq. 5. Table 5 summarises the results.

**TABLE 5:** Number of binding sites on albumin-based cryogels and biomolecule solutions.

	BSA-CG	OVA-CG	Am-BSA-CG	HEMA-BSA-CG	BSA solution	OVA solution
n	1.1 ± 0.3	1.65 ± 0.15	1.37 ± 0.5	1.45 ± 0.3	0.95 Makarska-Bialokoz, (2018)	∼1 Wang et al. (2013)

Free BSA in solution showed one single primary binding site during interaction with haemin in a solution (Makarska-Bialokoz, 2018). Wang and his team have shown that the binding site for OVA protein is one by studying the interaction of OVA with three purine alkaloids (caffeine, theophylline and diprophylline) (Wang et al., 2013). The deviation in the number of binding sites in this work with the reported number stemmed from the different operational conditions and the fact that protein molecules are crosslinked together and present a new binding interface inside the cryogel. Thus, comparing semi-solid cryogels with free protein solutions may be inaccurate.

## Conclusion

We designed and optimised albumin-based cryogels by crosslinking polymerisation at subzero temperatures. To increase the mechanical stability and improve the handling of the gel, BSA-CG was prepared inside a premade cryogel. Single-layer cryogels (OVA-CG and BSA-CG) demonstrated a higher affinity towards haemin (0.68 mg/ml of cryogel) than double-layer cryogels. In contrast, the double-layer cryogels have revealed superior mechanical stability with less adsorption capacity (0.47 and 0.59 mg/ml of cryogel for HEMA-BSA-CG and Am-BSA-CG, respectively). The number of binding sites of albumin-based cryogels with haemin was calculated to be between 1.1 and 1.65 for different cryogels.

The cryogelation technique is relatively straightforward and economical compared to other methods, such as membrane, yet practical for the mentioned goal. The possibility of autoclaving the network without destroying the macroporous configuration is another highlight of this material. However, increasing the mechanical strength and scaling up are common criticisms of cryogelation products. In this study, we tried to address the mechanical stabilities using a double-layer approach. Taking advantage of the interconnected channels of the cryogel to separate haemin from a particulate-containing fluid, e.g., blood and non-clarified crude, or using a different kind of crosslinking agent, are parts of ongoing research and will be addressed in future studies.

## Data Availability

The original contributions presented in the study are included in the article/Supplementary Material, further inquiries can be directed to the corresponding author.
