# Supplementary material for: Interaction of haemin with albumin-based macroporous cryogel: Adsorption isotherm and fluorescence quenching studies
Source: Front Bioeng Biotechnol. 2022 Nov 28;10:1072153. doi: 10.3389/fbioe.2022.1072153 (PMC9742477; doi:10.3389/fbioe.2022.1072153)
Supplement: Supplementary file 1 [file DataSheet1.PDF]

Supplementary information

Interaction of haemin with albumin-based macroporous cryogel: adsorption isotherm and fluorescence quenching studies

Solmaz Hajizadeh*, Cedric Dicko, Leif Bülow

Division of Pure and Applied Biochemistry, Department of Chemistry, Lund University,

22100, Lund, Sweden

*** Correspondance:**
Solmaz Hajizadeh
Solmaz.hajizadeh@tbiokem.lth.se

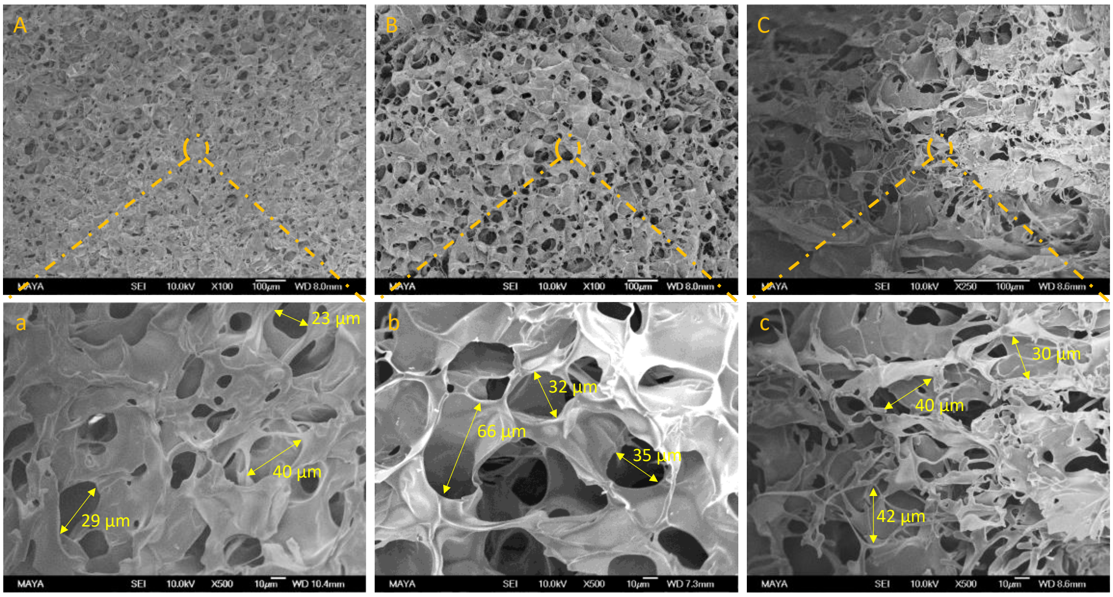

Figure S1. SEM images of BSA-CG A) BSA (30 mg/mL) and EDC (37 mg/mL); B) BSA (40 mg/mL) and EDC (37 mg/mL); C) BSA (50 mg/mL) and EDC (31 mg/mL). Images a, b and c are the high magnification of images A, B and C, respectively. The scale bars are 100 and 10 µm. The red arrows show the diameter of selected pores on the cryogel's cross-section measured by ImageJ.

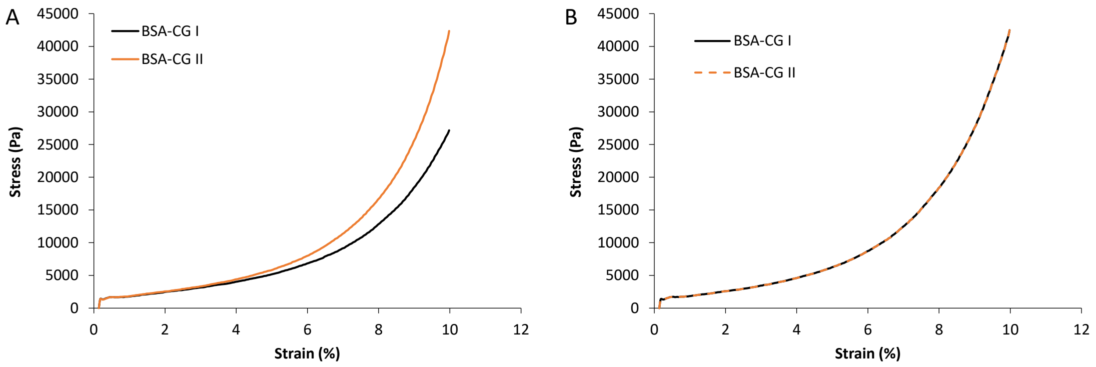

Figure S2. Stress plotted against strain (%) for BSA-CG (50 mg/mL protein concentration) up to 10% compression. A) EDC (56 mg/mL); B) EDC (46 mg/mL). BSA-CG I and II refer to two different wet cryogels in each group.

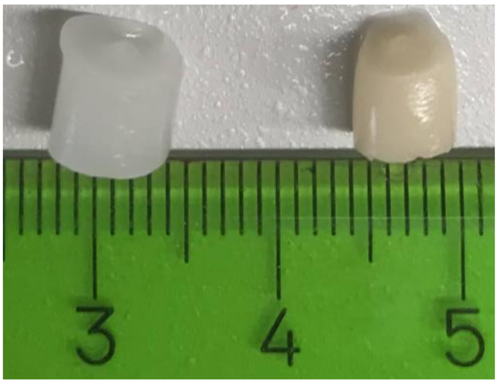

Figure S3. Digital photo of BSA-CG (250 µL). Left: before autoclaving and right: after autoclaving

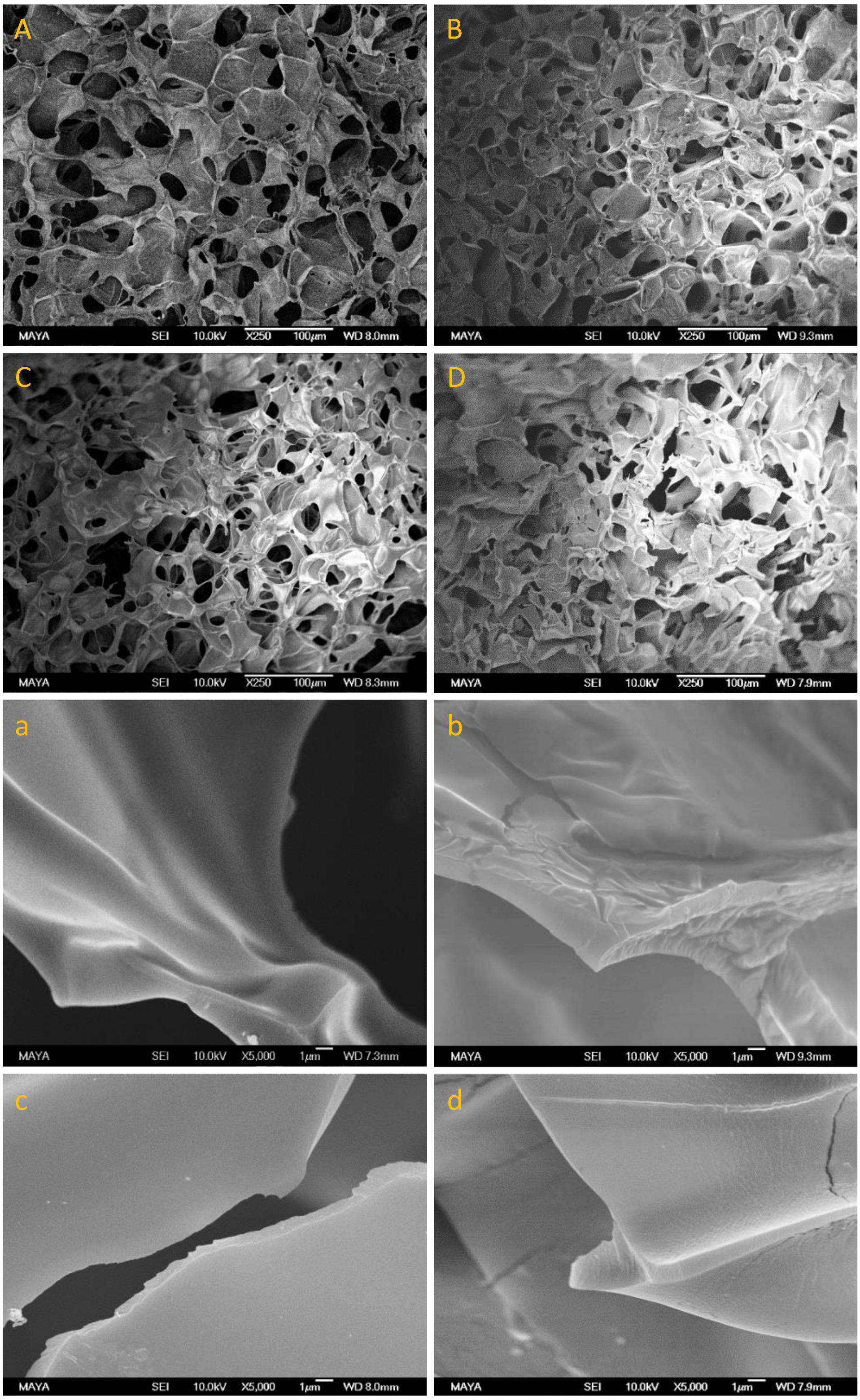

Figure S4. SEM images of albumin-based and control cryogels. A) BSA-CG before autoclaving; B) BSA-CG after autoclaving; C) Am-CG before autoclaving; D) Am-CG after autoclaving. Images a, b, c and d are the high magnification of images A, B, C and D, respectively. The scale bars are 100 and 1 µm.

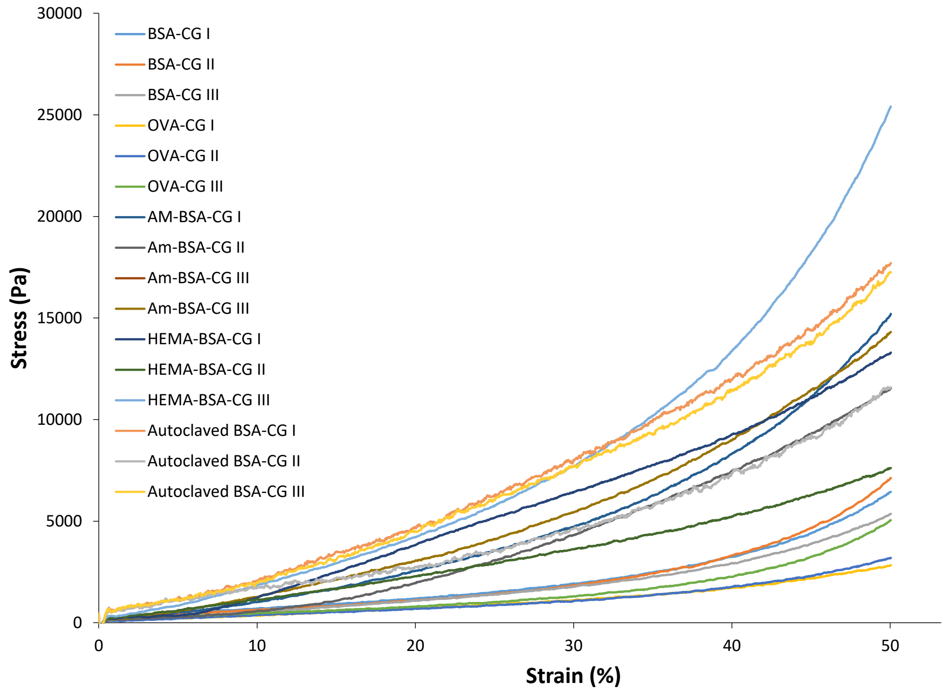

Figure S5. Stress plotted against strain (%). Each run was performed on three different wet cryogels in each group.

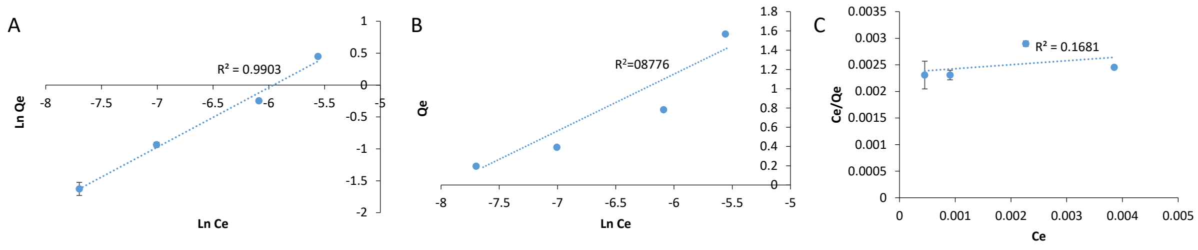

Figure S6. Fitting adsorption isotherm models with experimental data on haemin adsorption by BSA-CG at room temperature for 24 hours. A) Freundlich; B) Temkin and C) Langmuir models

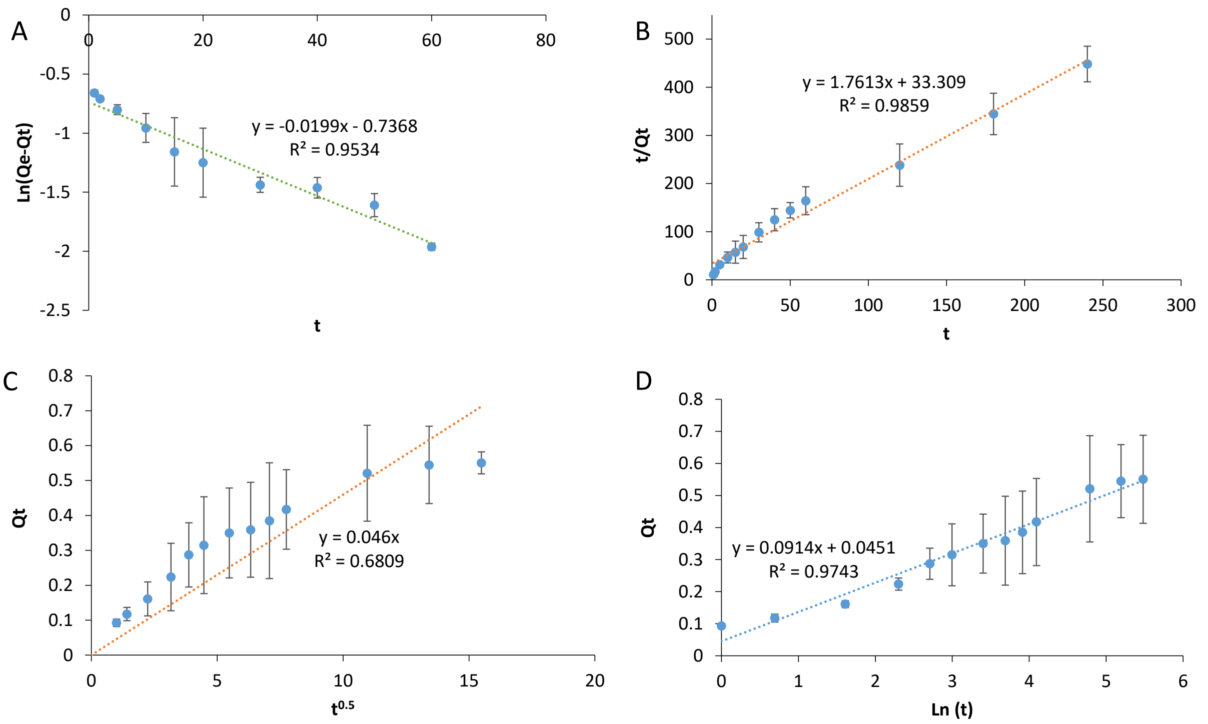

Figure S7. Fitting different models on the experimental data of haemin adsorption on BSA-CG with initial haemin concentrations of 0.2 mg/mL in 0.1 M phosphate buffer pH 7.4. A) Pseudo-first order; B) Pseudo-second-order; C) Intraparticle diffusion and D) Elovich.

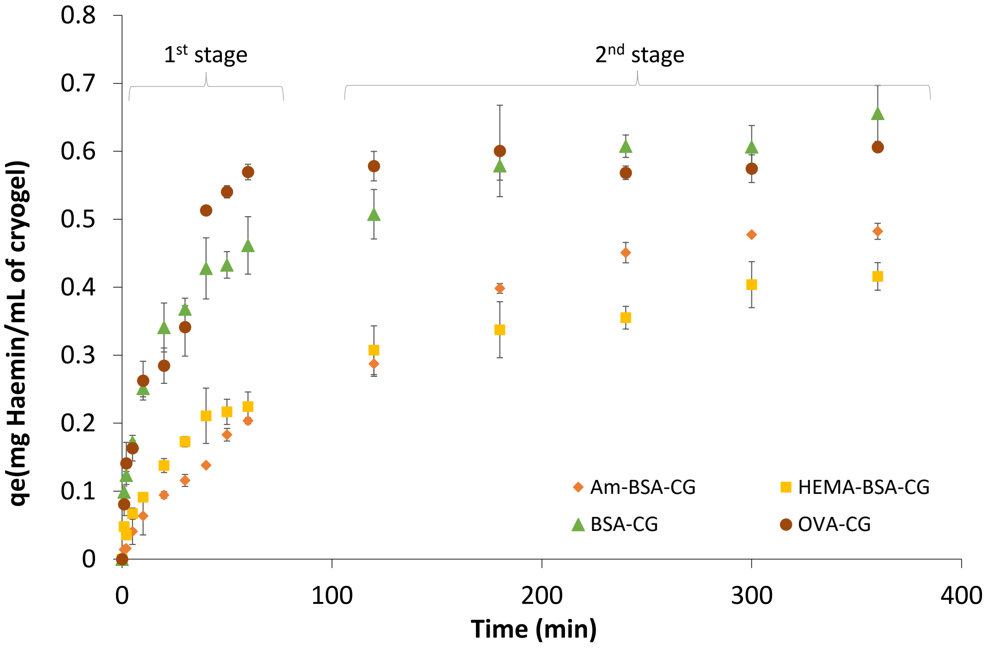

Figure S8. Kinetics isotherm of haemin adsorption with initial haemin concentrations of 0.2 mg/mL in 0.1 M phosphate buffer pH 7.4 for different cryogels between 0 and 400 minutes.

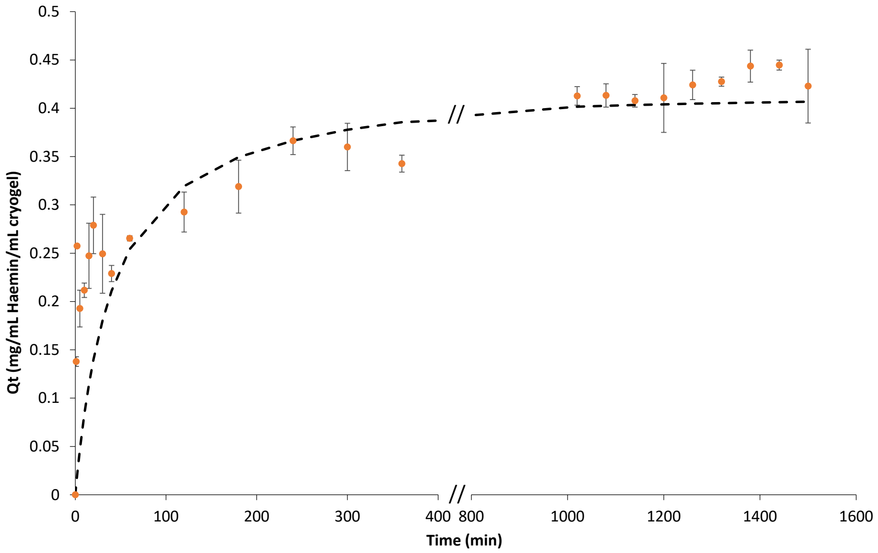

Figure S9. Fitting the experimental data of haemin adsorption with pseudo-second-order equation on autoclaved BSA-CG with initial haemin concentrations of 0.2 mg/mL in 0.1 M phosphate buffer pH 7.4. No data are available between 400-800 minutes.

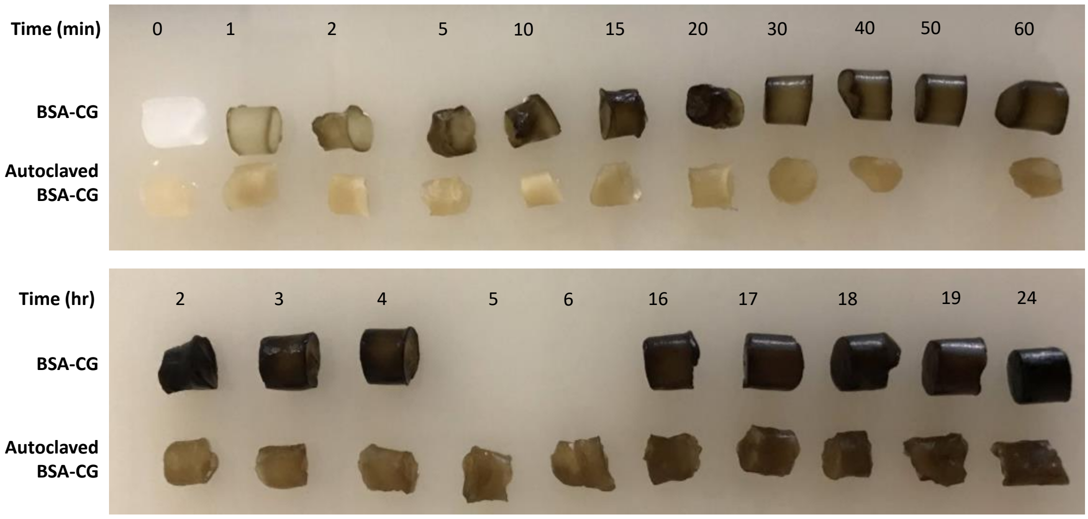

Figure S10. Digital image of BSA-CG (autoclaved and non-autoclaved) in contact with haemin solution (0.2 mg/mL in 0.1 M phosphate buffer pH 7) for different periods. No cryogel in positions 5, 6 and 50.

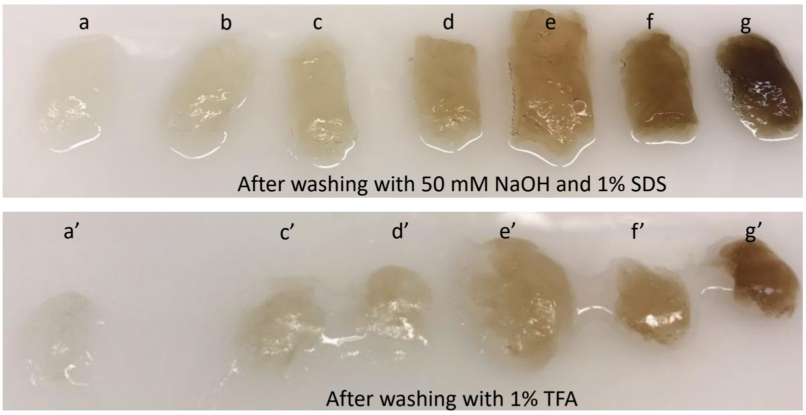

Figure S11. Digital image of the BSA-CGs, which were in contact with different haemin concentrations and then washed with NaOH (50 mM), SDS (1%) and trifluoroacetic acid (1%) separately in 3 steps. The initial concentration of haemin solutions before washing, a, a’) 0.004; b) 0.008; c, c’) 0.016; d, d’) 0.032; e, e’) 0.065; f, f’) 0.13 and g, g’) 0.26 mg/mL haemin in 0.1 M phosphate buffer pH 7.4.

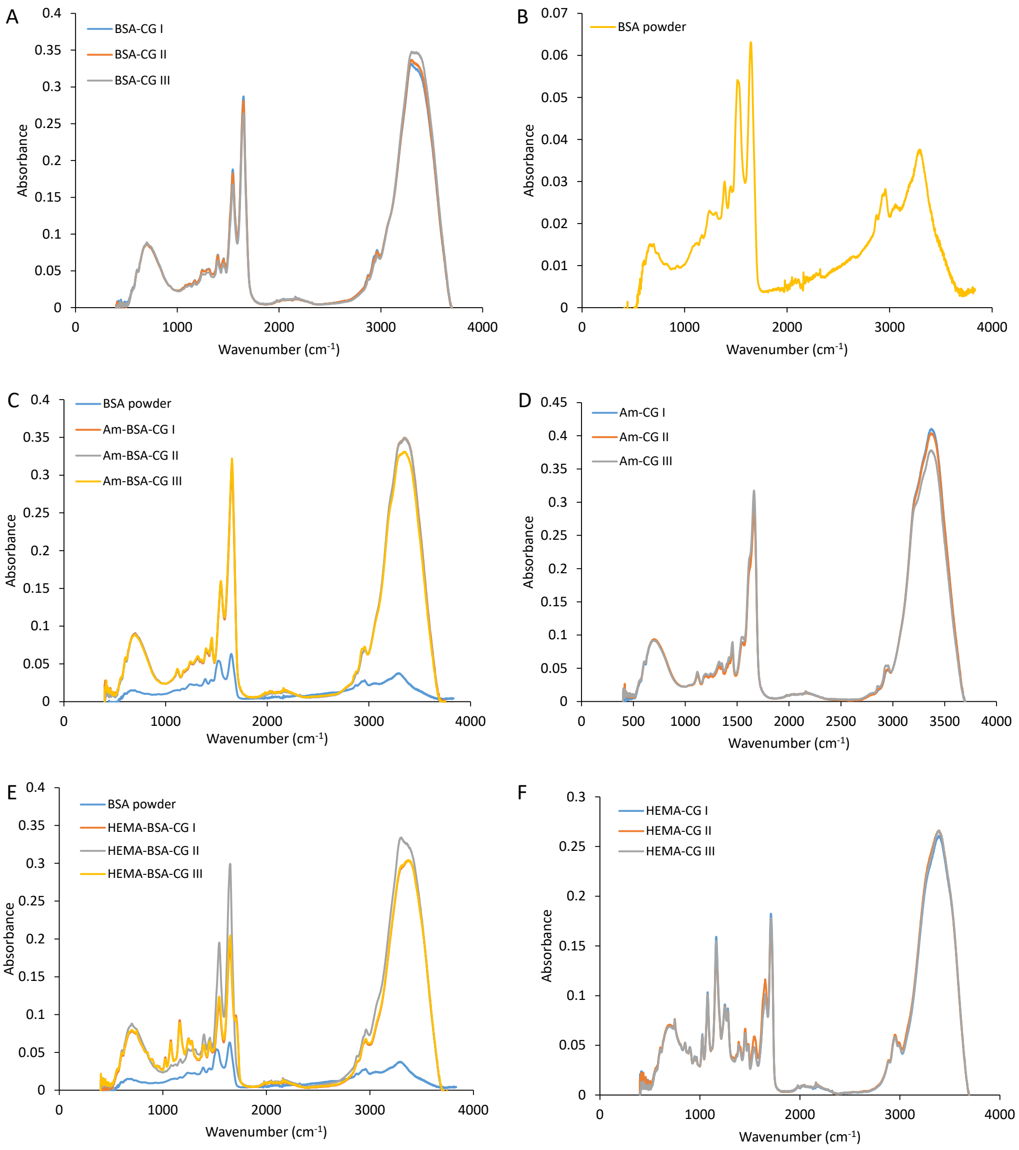

Figure S12. FTIR Spectra of A) BSA-CG, B) BSA powder, C) Am-BSA-CG, D) Am-CG, E) HEMA-BSA-CG, and F) HEMA-CG. I, II and III (the roman numerals) represent the top, middle and bottom sections of the cryogels, respectively.

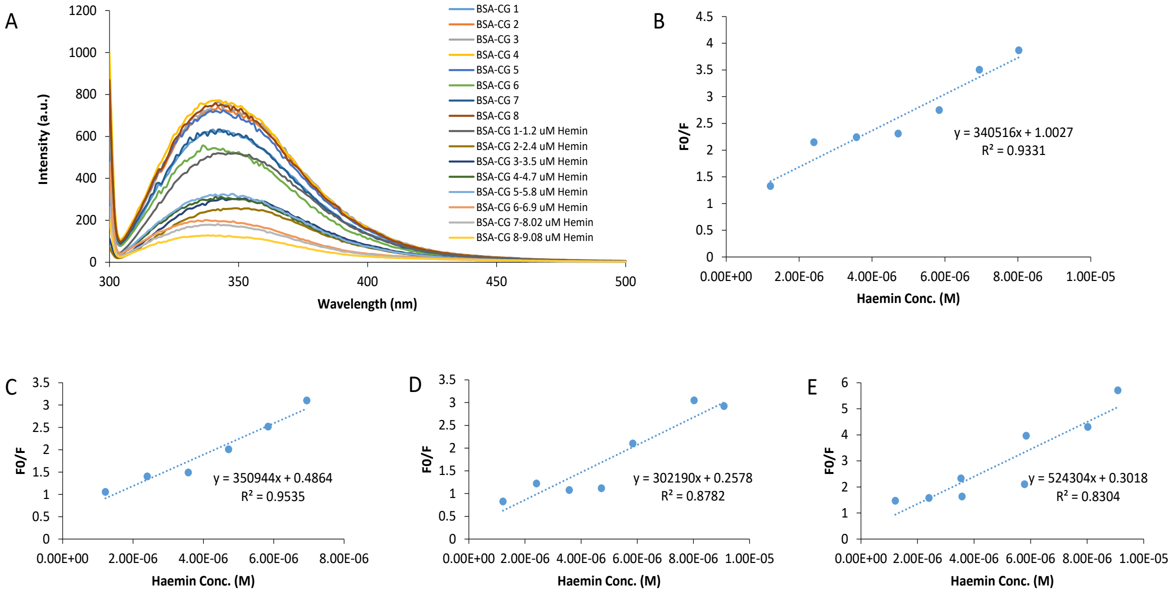

Figure S13. A) Fluorescence spectra of the interaction between the BSA-CG film and haemin at different concentrations (1.2 to 9 μM). BSA-CG 1-8 are different blank film cuts of the cryogel;

B-E) Stern-Volmer plots of haemin on BSA-CG, OVA-CG, Am-BSA-CG, and HEMA-BSA-CG.

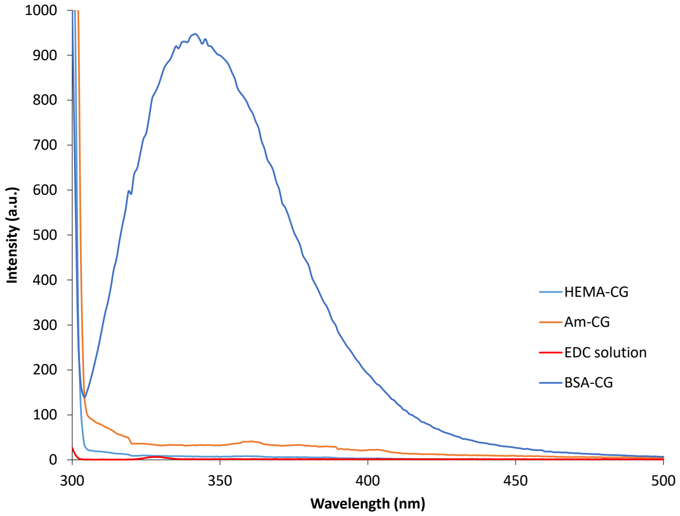

Figure S14. Fluorescence spectra of EDC solution, BSA-CG, Am-CG and HEMA-CG films in the absence of haemin and protein molecules
